# The role of gut microbiome in the complex relationship between respiratory tract infection and asthma

**DOI:** 10.3389/fmicb.2023.1219942

**Published:** 2023-07-27

**Authors:** Xiaoman Zhao, Mingge Hu, Huan Zhou, Yan Yang, Shiping Shen, Yannan You, Zheng Xue

**Affiliations:** Shanghai Municipal Hospital of Traditional Chinese Medicine, Shanghai University of Traditional Chinese Medicine, Shanghai, China

**Keywords:** gut microbiome, respiratory virus infection, asthma, short-chain fatty acid, interferon, vitamin D

## Abstract

Asthma is one of the common chronic respiratory diseases in children, which poses a serious threat to children's quality of life. Respiratory infection is a risk factor for asthma. Compared with healthy children, children with early respiratory infections have a higher risk of asthma and an increased chance of developing severe asthma. Many clinical studies have confirmed the correlation between respiratory infections and the pathogenesis of asthma, but the underlying mechanism is still unclear. The gut microbiome is an important part of maintaining the body's immune homeostasis. The imbalance of the gut microbiome can affect the lung immune function, and then affect lung health and cause respiratory diseases. A large number of evidence supports that there is a bidirectional regulation between intestinal flora and respiratory tract infection, and both are significantly related to the development of asthma. The changes of intestinal microbial components and their metabolites in respiratory tract infection may affect the occurrence and development of asthma through the immune pathway. By summarizing the latest advancements in research, this review aims to elucidate the intricate connection between respiratory tract infections and the progression of asthma by highlighting its bridging role of the gut microbiome. Furthermore, it offers novel perspectives and ideas for future investigations into the mechanisms that underlie the relationship between respiratory tract infections and asthma.

## 1. Introduction

Asthma is a common chronic respiratory disease in children, with a large number of morbidity and mortality worldwide (Johnson et al., 2021). To improve the prevention and treatment of asthma, it is crucial to understand the risk factors and underlying mechanisms that contribute to asthma's onset and exacerbation. Patients with asthma may experience an acute exacerbation of symptoms and loss of control of the disease, even with appropriate management, which is usually triggered by respiratory infections (Fujitsuka et al., 2011; Choi et al., 2018). Respiratory viral infections are the most common cause of asthma exacerbation throughout childhood and beyond (Johnston et al., 1996; Zheng et al., 2020; Chen et al., 2023). There is significant evidence suggesting that respiratory infections play a role in the development of asthma, but the underlying mechanisms have yet to be fully elucidated (Busse et al., 2010; Jackson and Gern, 2022).

The gut microbiome is the most abundant microbial community in humans and mice, consisting of ~4 × 10^14^ species of bacteria that have coevolved with the host immune system to establish a symbiosis. The diverse range of gut bacteria is crucial for maintaining the host's immune balance and is associated with the development of various diseases, including asthma, viral infections, and other respiratory diseases (Eckburg et al., 2005). Multiple clinical studies and animal experiments have confirmed the correlation between gut microbiota and respiratory infections, as well as asthma.

Arrieta et al. (2015) found that infants at risk of asthma exhibited transient changes in their gut microbiome composition during the first 100 days of life, including decreased relative abundance of Lachnospira, Veillonella, Faecalibacterium, and Rothia. Supplementing these bacterial groups notably reduced airway inflammation in adult offspring of germ-free mice. Moreover, there was a significant negative correlation between Lachnospira and cellular counts in the bronchoalveolar lavage fluid (BALF) of mice, which strongly suggested that the presence of Lachnospira reduced lung inflammation. These findings indicate that those bacterial taxa may not only be correlated with asthma susceptibility but also play a causal role in preventing asthma development. Gut microbial dysbiosis has also been found in respiratory infections, especially in respiratory syncytial virus (RSV) infection and influenza virus infection (Wang et al., 2014; Groves et al., 2018).

**Figure 1:**
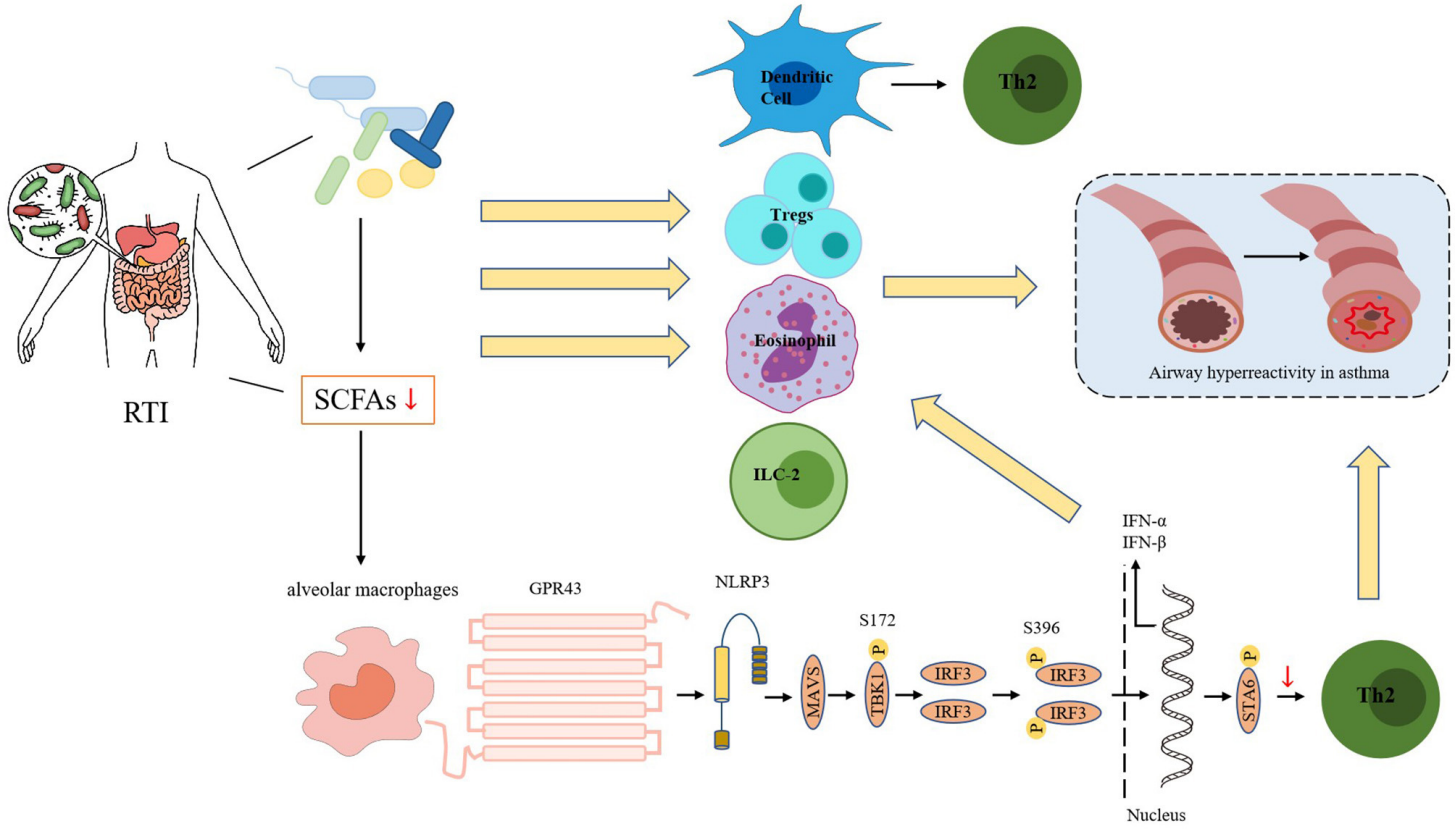
Respiratory tract infections can alter the microecology of the intestines, leading to changes in the abundance of gut microbiome and a reduction in bacteria that produce short-chain fatty acids (SCFAs). So there is a decrease in SCFA production. The reduction in SCFA levels can impact the function and fate of various immune cells, including dendritic cells (DCs), regulatory T cells (Tregs), eosinophils, and type 2 innate lymphoid cells (ILC2s). Additionally, the SCFA receptor GPR43 can interact with the NOD-like receptor protein 3 (NLRP3) to promote the aggregation and signal transduction of the mitochondrial antiviral signaling protein MAVS. When acetate binds to GPR43, it enhances MAVS aggregation, activates downstream TBK1/IRF3, and promotes the production of interferon-I (IFN-I). IFN-I can increase the number of Th2 cells and eosinophils, which can lead to airway hyperresponsiveness in asthma.

Resident microbes in the gut can use dietary fiber to produce short-chain fatty acids (SCFAs), thereby regulating the host immune response (Silva et al., 2020). Brestoff and Artis (2013) and Niu et al. (2023) suggest that SCFAs can play a pivotal role in regulating asthma by modulating the differentiation and function of immune cells. In addition, SCFAs can improve the production of interferons (IFNs) to enhance the respiratory immune system's ability to combat viral pathogens (as shown in Figure 1).

Based on the role of respiratory infections in the pathogenesis of asthma, these results suggest that intestinal microbial dysbiosis may play a role in infection-induced and aggravated asthma, but the mechanism is still unclear. In addition, vitamin D levels were inversely associated with asthma severity, including hospitalizations for asthma with severe infections (Brehm et al., 2009). At the same time, there is a clear association between VD deficiency and viral respiratory infections, and there is an inverse association between vitamin D supplementation and rhinovirus infections (Eroglu et al., 2019; Jartti et al., 2021). Notably, Jones et al. (2013) showed that gut microbiota can alter gut vitamin D metabolism (VDM) and that probiotic supplements can affect VD levels in circulation. Therefore, gut microbial dysbiosis may lead to changes in vitamin D levels, affect the occurrence of respiratory viral infections, and then affect the severity of asthma. In this article, we review the gut microbiota-related potential mechanisms of how respiratory infections affect the development and exacerbation of asthma, including the relationship between the gut microbiota and respiratory viral infections and how the gut microbiota regulates the host immune response in the pathogenesis of asthma.

## 2. Relationship between gut microbiome and susceptibility to asthma, and characteristics of gut microbiota in asthma

Generally, the gut microbiota of healthy adults consists mainly of Firmicutes (including Roseburia, Enterococcus, and Faecalibacterium) and Bacteroides (such as Bacteroides and Prevotella; Costea et al., 2018; Rinninella et al., 2019; Gomaa, 2020; Illiano et al., 2020). This diverse microbiota is rich in microorganisms that can ferment substances which is hard to decompose such as dietary fiber to produce SCFAs, including butyrate, propionate, and acetate. These SCFAs can bind to host-derived cytokines and chemokines, translocate through the blood and lymphatic systems, and influence lung immunity (Parada Venegas et al., 2019).

Dysregulation of the gut microbiome and reduced metabolic capacity can impair local and pulmonary immunity, thereby increasing susceptibility to lung diseases such as asthma and respiratory viral infections. Epidemiological studies support the connection between gut microbiota dysbiosis in early life and an increased risk of childhood asthma, while early exposure to dogs, farm life, and microorganisms is negatively associated with the risk of wheezing disease (Lynch et al., 2014; Ludka-Gaulke et al., 2018). In fact, compared to non-asthmatic children, school-age children with asthma exhibited lower gut microbiome diversity before one month of age (Abrahamsson et al., 2014). Another study found significant differences in the relative abundance of gut bacteria between asthma and healthy children. Dialister, Faecalibacterium, and Roseburia of Firmicutes were significantly reduced in children with asthma (Chiu et al., 2019), which may increase susceptibility to viral infections and contribute to the development of asthma.

The differences in gut microbiota between asthma and non-asthma include not only the diversity and relative abundance of intestinal microorganisms; but also the intestinal metabolites, such as amino acids, butyrate, and other SCFAs. These metabolites are produced by certain intestinal microorganisms. Bacteroidetes and Firmicutes are the dominant bacterial phyla-producing SCFAs, both of which can produce acetate. Butyrate is mainly produced by Ruminococcus, Clostridium, and Coprococcus in Firmicutes. Propionate is produced by Bacteroidetes such as Prevotella and Bacteroidetes. Chiu et al. (2019) discovered that butyrate in stool was decreased in children with asthma and negatively correlated with serum IgE levels. This suggests that reduced butyrate production and the resulting imbalance in intestinal epithelial barrier function may promote the uptake of allergens in allergic asthma. The study also found that children with asthma exhibited reduced populations of butyrate-producing bacteria (including Coprococcus and Roseburia) and increased Clostridium, which was negatively associated with butyrate. This suggests that butyrate reduction is associated with specific gut microbiota changes. Similar alterations in gut microbiota have also been observed in adults with asthma. Fedosenko et al. (2014), Masik et al. (2019), Zolnikova et al. (2020), and Ozimek et al. (2022), have observed changes in gut microbiota in adult patients with bronchial asthma, including an increase in the proportion of Proteobacteria and a decrease in bacteria that can produce butyrate and acetate. The changes in gut microbiota in asthma mainly include the decrease of gut microbial diversity, the decrease of the relative abundance of beneficial microorganisms (such as Faecalibacterium), and the reduction or even depletion of SCFAs-producing bacteria.

The gut microbiota has been shown to impact pulmonary diseases such as asthma via the lung-gut axis, which has led to the possibility of probiotic therapy. A recent study found that supplementing with Lactobacillus rhamnosus reduced the incidence of childhood asthma, likely due to the probiotics altering the composition of the microbiome and the levels of SCFAs (Cereta et al., 2021). In another study, researchers discovered that two types of Lactobacillus decreased airway inflammation in mice with HDM-induced allergic asthma, inhibited Th2 and Th17 immune responses, and promoted Treg responses. This was accompanied by changes in the heterogeneity, composition, and metabolism of the intestinal microbiome (Li et al., 2020). It has been demonstrated that dietary fiber supplementation can have anti-inflammatory effects in asthma. And this is attributed to the increased production of SCFAs resulting from the fermentation of dietary fiber by gut bacteria (Trompette et al., 2014). Cait et al. (2017) found that the use of vancomycin reduced the intestinal flora that produces SCFAs, which increased the susceptibility of mice to OVA-induced asthma and papain-induced pulmonary inflammation. In comparison to the control group, supplementation with SCFAs was found to reduce the vancomycin-induced enhancement of lung inflammation in asthmatic mice. These SCFAs can exhibit anti-inflammatory effects by activating free fatty acid receptors, such as G protein-coupled receptors 41 and 43 (GPR41 and GPR43), in patients with asthma and animal models of asthma (Halnes et al., 2017).

Trompette et al. (2014) found that SCFAs like propionate could impair the ability of DC to activate Th2 effector cells in the lung, making allergic airway inflammation unsustainable. SCFAs can also regulate the function of various immune cells, such as type 2 innate lymphoid cells (ILC2; Thio et al., 2018), regulatory T cells (Tregs; Arpaia et al., 2013), and inhibit an increase of eosinophils (Theiler et al., 2019), thereby reducing airway hyperresponsiveness in asthma. Detailed mechanisms are described in Section 3 below. Additionally, SCFAs can prevent and restore impaired airway epithelial barrier function. Epithelial barrier dysfunction is a characteristic and pathological mechanism of airway inflammation in asthma patients (Georas and Rezaee, 2014). In comparison to non-asthmatic patients, the bronchial epithelial barrier function in asthmatic patients is impaired (Inoue et al., 2020), making it easier for pathogens or allergens to enter the airway tissue, activating an immune response, and leading to asthma exacerbation (da Silva, 2021). Richards et al. (2020) demonstrated that propionate and butyrate can enhance the barrier function of human bronchial epithelial cells and restore the impaired barrier function induced by stimulation with IL-4, IL-13, and dust mite extract. This is due to the increased expression of ZO-1, one of the tight junction proteins, by SCFA. In summary, studies have demonstrated that SCFAs exert a protective effect against asthma, indicating that a decrease in SCFAs may exacerbate asthma symptoms (Thio et al., 2018).

## 3. Changes of gut microbiome and its derivatives after pulmonary infection and the mechanism of these changes

Groves et al. (2018), Lv et al. (2021), and Zhu et al. (2021) have shown that pulmonary viral infections can modify the composition of intestinal flora, although the underlying mechanism remains unclear. The changes in gut microbiome in mice with asthma after respiratory syncytial virus (RSV) infection were studied for the first time. The results showed that the Bacteroidetes increased and the Firmicutes decreased in the intestinal microbiota of mice after RSV infection (Lu et al., 2020). The loss of balance between the two is associated with many diseases or disorders (Turnbaugh et al., 2006). Additionally, a reduction in the Lactobacillus family was also found in this study. This is consistent with changes in the Lactobacillus family following influenza virus infection (Wang et al., 2014). Alterations in the gut microbiota following respiratory infections have also been observed in humans. A study on recurrent respiratory tract infections (RRTI) (Li et al., 2019) found that Verrucomicrobia and Tenericutes were significantly reduced in RRTI patients compared to healthy volunteers. A systematic review of 11 published studies investigating changes in the gut microbiome of patients with confirmed or suspected respiratory tract infections (Woodall et al., 2022) revealed that Firmicutes, Lachnospiraceae, Ruminococcaceae, and Ruminococcus were reduced, while Enterococcus was abundant compared to the healthy control group. Enterococcus has been identified as an opportunistic pathogen causing community-acquired and nosocomial infections (Ager and Gould, 2012; Yu et al., 2015). These altered intestinal bacteria can produce SCFAs through the fermentation of dietary fiber. The above studies show that respiratory infections may lead to a decrease in SCFAs. Evidence suggests that RTI-induced changes in the gut microbiome can lead to the depletion of beneficial gut bacteria that produce SCFAs, such as Lachnospiraceae, which belongs to Firmicutes and can ferment pectin and fructose to produce acetate, propionate, and ethanol (Boutard et al., 2014). Reduced levels of SCFAs affect the immune system and aggravate asthma (Trompette et al., 2014; Cait et al., 2017). SCFA can promote the production of interferon, thereby inhibiting respiratory virus replication and promoting virus clearance (Ji et al., 2021). Based on the multiple effects of SCFAs mentioned earlier in the article, it is suggested that a decrease in the beneficial SCFAs caused by respiratory infections may weaken their protective effect against asthma.

In general, respiratory virus infections lead to changes in the composition of intestinal flora, including an increase in Bacteroidetes, a decrease in short-chain fatty acid-producing bacteria such as Firmicutes and Pilospirillum, and an increase in opportunistic pathogens such as Enterococcus (Boutard et al., 2014; Groves et al., 2018; Woodall et al., 2022). These changes are similar to the changes observed in the gut microbiota of individuals with asthma, indicating that an imbalance in gut microbiota may contribute to the development and exacerbation of asthma following respiratory infections. An animal study (Lu et al., 2020) showed that the gut microbiota of asthmatic mice infected with RSV was altered, and this was correlated with elevated Th2 cytokine levels and increased asthma severity. Furthermore, the gut microbiome can influence the development of asthma by affecting the early maturation of the immune system in response to viral infections, which may be linked to a reduction in SCFAs (Lynch et al., 2017). To summarize, the changes in gut microbiota following respiratory virus infections have implications for the pathogenesis and exacerbation of asthma and warrant further investigation.

Although many studies have confirmed changes in the microbiome and SCFA levels following lung infection, the functional implications and underlying mechanisms of these changes are not yet clear. Both type I and II interferons (IFNs) have been implicated in the alteration of intestinal microbiota caused by influenza virus infection. Deriu et al. (2016) knockout the mouse IFN-I gene and found that influenza virus pulmonary infection can significantly alter the distribution of intestinal microbiota through IFN-I-dependent mechanism, leading to the consumption of anaerobic bacteria and the enrichment of proteobacteria in the intestine. It is important to note that Proteus expansion is almost always accompanied by a decrease in the abundance of butyrate-producing bacteria, which is characteristic of microbial dysregulation. Wang et al. (2014) found that influenza infection mediated changes in intestinal microbiome composition through IFN-γ production by pulmonary CCR9^+^CD4^+^ T cells recruited in the small intestine. Another factor that may cause these changes is mucus. Mucus not only protects against exposure to microbes but also acts as an energy source for certain bacteria. If the mucus composition changes, microorganisms can benefit ecologically by utilizing it (Derrien et al., 2010). Usually, the mucin Muc5ac is expressed in the airway but not in the colon (Koeppen et al., 2013). Groves et al. (2018) observed elevated levels of Muc5ac in the colon of RSV-infected mice and suggested that Muc5ac could be linked to dysbiosis of intestinal flora. However, whether the increase in mucin is due to viral infection stimulating Muc5ac expression in colonic goblet cells remains to be investigated. Similar conditions have been observed elsewhere, where changes in the composition of the vaginal flora have been associated with increased levels of Muc5B and Muc5ac in the cervix (Borgdorff et al., 2016). However, the effect of mucus on gut flora has not been studied previously.

Loss of appetite caused by infection is another explanation for the observed changes in microbiome composition after a lung infection. The similarity in gut microbial changes after RSV and influenza virus infections suggests the presence of common underlying mechanisms based on pathogen-specific immunity for viral infections. Weight loss is a common symptom in mice after both infections, associated with reduced food intake. Loss of appetite has also been reported in humans infected with influenza virus and respiratory syncytial virus (Monto et al., 2000; Widmer et al., 2012). Diet is the largest factor in microbiota composition, and studies have shown that reduced caloric intake in mice and humans is associated with significantly increased abundance ratios of Bacteroides and Firmicutes, which is consistent with changes in intestinal microbiota composition in mice and humans with respiratory infections.

Another study by Groves et al. (2018) found that weight change after viral infection was not related to viral load but to CD8^+^ T cells. Depletion of CD8^+^ T cells during respiratory syncytial virus infection could reduce loss of appetite and reverse changes in gut microbiome. Although the mechanism of how CD8^+^ T cells affect appetite is still unclear, it is likely mediated by cytokines or chemokines produced by CD8^+^, such as IL-6 and tumor necrosis factor-β (TNF-β).

## 4. The role of gut microbial changes caused by respiratory infection in the development and exacerbation of asthma

### 4.1. The reduction of SCFAs can contribute to asthma exacerbations

**Figure 2:**
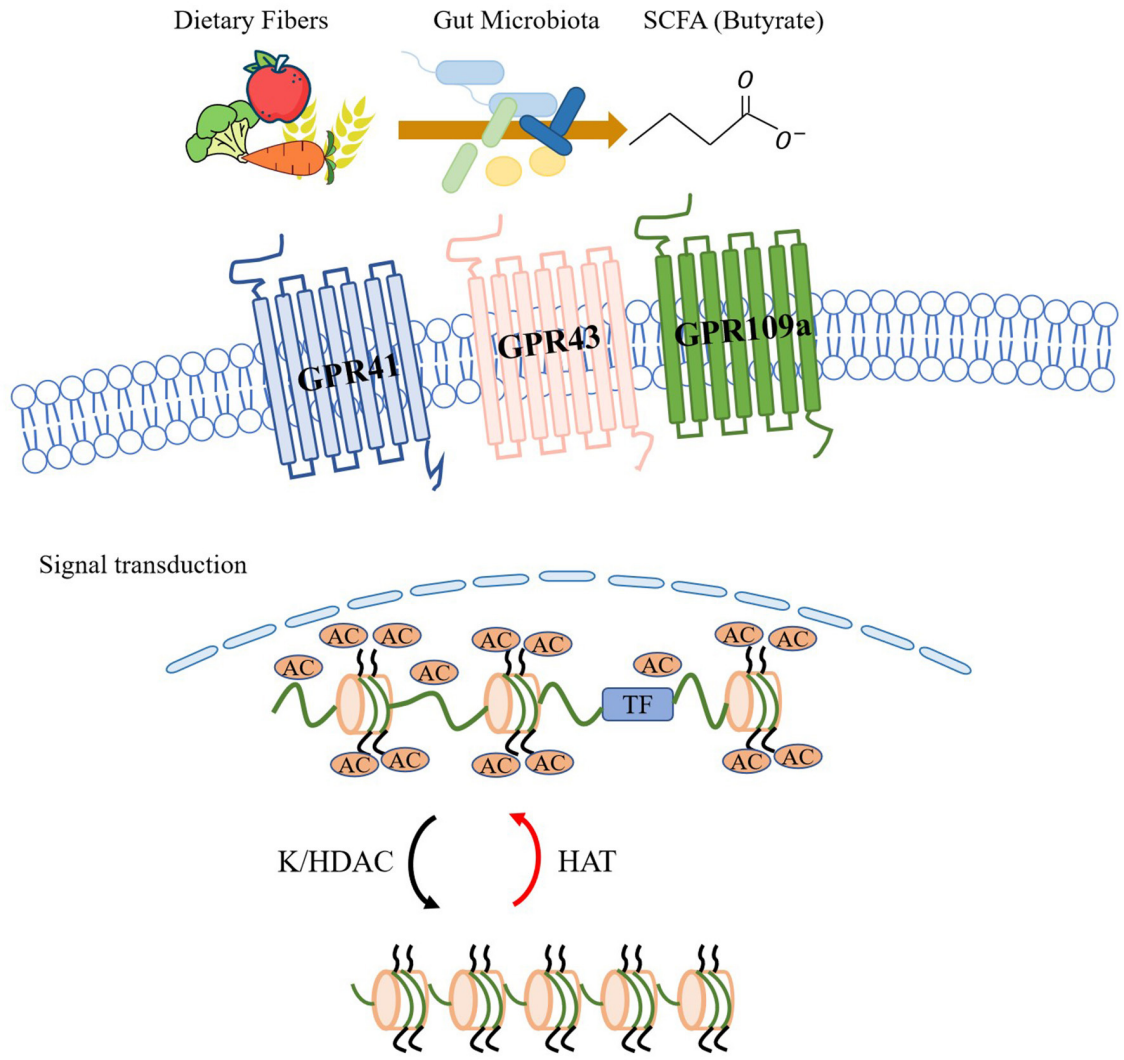
The gut microbiome has the ability to utilize dietary fiber to produce short-chain fatty acids (SCFAs) like acetate. These SCFAs can activate G protein-coupled receptors (GPCRs) such as GPR43, GPR41, and GPR109a, and initiate signal transduction. SCFAs also exert significant inhibitory effects on lysine/histone deacetylase (K/HDAC) activity. Through their HDAC inhibitory effects and histone acetyltransferase (HAT) promoting effects, SCFAs increase histone acetylation, which in turn promotes histone translational modifications and regulates systemic immune responses.

The above sections of the article suggest that respiratory infections can alter the gut microbiome, leading to a reduction in protective gut bacteria that produce SCFAs. This reduction in SCFA-producing bacteria may contribute to a diminished protective effect of SCFAs in asthma, potentially leading to the aggravation of symptoms. The role of SCFAs in the pathogenesis of asthma has been extensively studied in the context of the lung-gut axis, particularly in children. SCFAs have been shown to regulate the systemic immune response by activating GPCRs or inhibiting histone deacetylases (HDACs), leading to downstream signal transduction (as shown in Figure 2). In their study, Halnes et al. (2017) demonstrated that SCFAs can upregulate the gene expression of GPCRs, including GPR41 and GPR43, which can alleviate airway inflammation in asthma. Furthermore, SCFAs can inhibit HDACs, a class of proteases present in eukaryotic cells that play an important role in maintaining glucocorticoid sensitivity, regulating airway inflammation, airway remodeling, and airway hyperresponsiveness (AHR). Yip et al. (2021) have shown that inhibition of HDAC activity can effectively reduce respiratory diseases in mice. Butyrate, among SCFAs, is the most potent inhibitor of HDAC activity and can directly inhibit HDAC in a dose-dependent manner (Kaiko et al., 2016).

**Figure 3:**
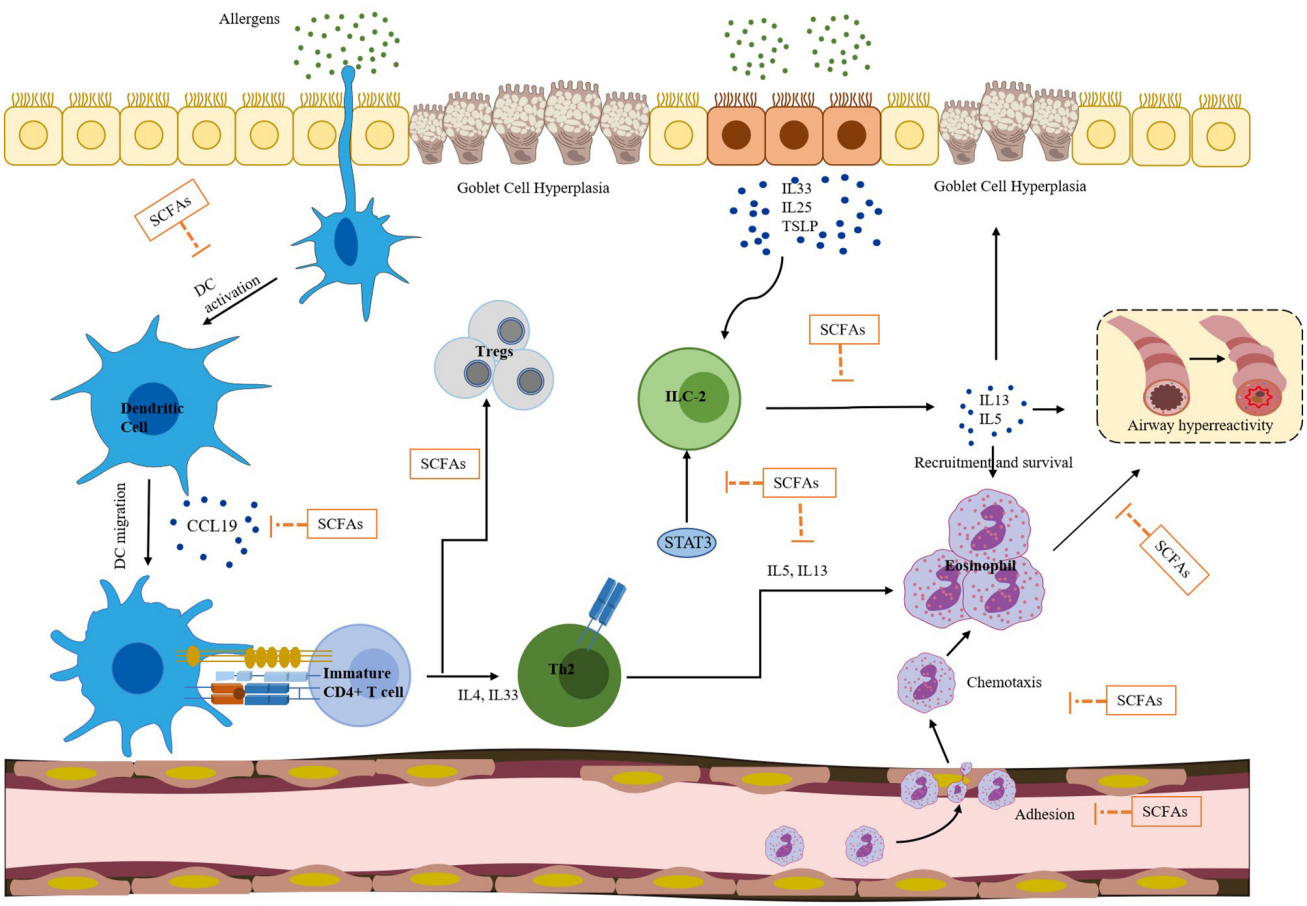
SCFAs have a broad spectrum of effects on immune cells involved in allergic asthma, which is a complex inflammatory disease. Exposure to allergens can result in eosinophilia, airway hyperresponsiveness, and goblet cell hyperplasia, all of which are driven by a combination of dendritic cells (DCs), Th2 cells, ILC2s, and eosinophils. SCFAs can inhibit DC activation and migration, which can stimulate the differentiation of immature/naive CD4^+^ T cells into the Th2 lineage. Additionally, SCFAs reduce the responsiveness of DCs to CCL19, inhibiting the migration of DCs. SCFAs can promote the transformation of naive CD4^+^ T cells from Th9 to regulatory T cells (Tregs) and inhibit ILC2 secretion of IL-5 and IL-13 cytokines. IL-5 can activate eosinophils, and IL-13 can stimulate airway epithelial cells to produce mucus and aggravate airway hyperresponsiveness. Furthermore, SCFAs can down-regulate the expression of GATA3, a key transcription factor involved in ILC2 development and function. SCFAs can also decrease the adhesion, migration, and survival of human eosinophils. Overall, SCFAs play a significant role in regulating the differentiation and function of various immune cells, and thus have the potential to improve allergic asthma.

SCFAs can also regulate the activity of ILC2, Tregs, and DCs, and inhibit eosinophilia through the signal transduction pathways mentioned above. Figure 3 illustrates various effects of SCFAs on immune cells in allergic asthma. These cells are closely associated with Th2-mediated immune responses, which are considered a core molecular mechanism of asthma. ILC2 can produce Th2-type cytokines, such as IL-5 and IL-13, leading to eosinophilia and AHR. A research (Thio et al., 2018) has demonstrated that butyrate can play a crucial role in regulating the proliferation and function of ILC2. The administration of butyrate can inhibit the production of IL-13 and IL-5 and significantly alleviate ILC2-driven AHR and inflammation. Moreover, Lewis et al. (2019) reported that butyrate can down-regulate the expression of GATA3, a key transcription factor involved in ILC2 development and function. This down-regulation effectively suppresses ILC2-driven AHR. SCFAs can induce the differentiation of Tregs by inhibiting HDACs and stimulating GCRP, including GPR43, GPR41, and GPR109a (Arpaia et al., 2013). This process may be related to the suppression of allergic airway diseases. Hu et al. (2021) have shown that supplementation with certain gut microbiota or SCFAs can increase the proportion of Tregs in house dust mite-induced allergic asthma. The CCL19/21 and CCR7 chemokine axes play a crucial role in mediating DCs migration from inflamed tissues to collected lymph nodes. DCs isolated from mice treated with butyrate respond less efficiently to CCL19 compared to the group administrated with vancomycin alone, indicating that butyrate attenuates CCL19-induced DCs migration (Cait et al., 2017). Additionally, vancomycin treatment significantly increases the number of DCs in the mediastinal lymph nodes of mice, while exogenous SCFAs reverse this increase and reduce the number of DCs to the control level. This suggests that SCFA exposure can alter the migration behavior of DCs. Furthermore, Kim and Lee (2017) have demonstrated that HDAC inhibitors (HDACis) such as trichostatin A (TSA), sodium butyrate, and scriptaid strongly inhibit the migratory activity of DCs. The inhibitory activity of HDACis is caused by a decrease in CCR1 expression on the cell surface and inhibition of phosphorylation of p38 mitogen-activated protein kinase (MAPK), extracellular signal-regulated kinases 1 and 2 (ERK1/2), and c-Jun N-terminal kinase (JNK). Numerous studies have shown that SCFAs can effectively inhibit HDACs, with butyrate being a known class I and II HDAC inhibitor (Hamminger et al., 2020), and propionate also having this inhibitory effect (Song et al., 2022). Studies have shown that butyrate can strongly inhibit HDAC in CD8^+^ T cells, and TSA has a similar effect on CD8^+^ T cells (Luu et al., 2018). Theiler et al. (2019) used experiments *in vitro* and *in vivo* to demonstrate that butyrate can reduce the adhesion, migration, and survival of human eosinophils, and these effects are independent of GPR41 and GPR43. Instead, they are accompanied by histone acetylation and can be mimicked by HDAC inhibitors. This suggests that butyrate can inhibit eosinophil accumulation, reduce the level of type 2 cytokines, improve AHR in asthmatic mice, and may have a causal relationship with HDAC inhibition. Overall, SCFAs can regulate the systemic immune response and reduce AHR in asthma by inhibiting the Th2 response through direct effects on ILC2, T cells, DCs, and eosinophils.

### 4.2. Interferon deficiency regulated by gut microbiome may contribute to the exacerbation of asthma

IFN is one of the inflammatory mediators induced after viral infection. It plays a crucial role in initiating a robust antiviral response, which includes impairing viral replication and transmission between cells and enhancing innate and adaptive immune responses against viruses and some bacteria. IFNs can be classified into three types: IFN-1 (IFN-α and IFN-β), IFN-2(IFN-γ), and IFN-3 (IFN-λ family, including IFN-λ1, IFN-λ2, and IFN-λ3). IFN-1 and IFN-3 are the primary IFN isoforms induced by pulmonary viral infections. In contrast, IFN-2 (IFN-γ) is typically induced by cytokines and primarily expressed by immune cells such as T cells, NK cells, or monocytes, rather than in response to viral induction (Bar Yoseph et al., 2015).

Previous studies have found that patients with asthma may have impaired production of type I and III IFNs, which could increase their susceptibility to viral infection (Confino-Cohen et al., 2014). Plasmacytoid dendritic cells (pDCs) are the primary cells responsible for producing IFN-α against viruses. Lin et al. (2020) found that after RV stimulation, interferon-α production by pDCs was significantly increased in normal subjects but not in asthmatic patients. This provides further evidence of a defective innate response to RV infection in asthmatic patients. Moreover, evidence shows that both asthmatic patients regardless of atopic status, and atopic patients without asthma have an insufficient interferon response to RV infection during childhood (Baraldo et al., 2012). However, Sykes et al. (2013) specifically selected subjects with well-controlled asthma in another study and did not observe any defects in the induction of IFN by RV. Although these results show some variability, most studies suggest that patients with asthma have an insufficient interferon response. Interestingly, type I IFN production has been shown to be promoted by SCFAs, which are typically reduced in respiratory infections and asthma. This suggests that the impaired expression of type I IFN in asthma may be associated with a reduction in SCFAs.

#### 4.2.1. The mechanisms by which SCFAs regulate the generation of interferons

Growing evidence suggests intestinal malnutrition can impact the immune response of the lungs (Kulas et al., 2019). Additionally, changes in gut microbes reported in respiratory viral and bacterial infections have been linked to immune inflammation in the lungs. Studies have shown that lung infections can cause inflammatory cell infiltration and increased levels of intestinal IFN-γ and interleukin 17 (IL-17). Furthermore, a decrease in the diversity of intestinal bacterial microbiota has been observed early in the infection (Kulas et al., 2019). These findings suggest that infections can disrupt the balance of gut microbes, leading to impaired interferon production and asthma exacerbation.

Increased production of IFNs can enhance the body's ability to prevent respiratory virus infections and clear viruses, thereby reducing the exacerbation of asthma caused by viruses. Studies have shown that improving intestinal microecology can reduce viral load, increase the expression of IFN-α, IFN-γ, and IL-1β, and reduce TNF-α (Wang et al., 2021). The specific mechanism underlying these effects is still unclear, but it may be related to the role of SCFAs in modulating the immune response. A study (Ji et al., 2021) demonstrated that administering probiotics can significantly increase the abundance of SCFAs-producing bacteria in mice infected with RSV, resulting in higher levels of SCFAs in the serum. Furthermore, it was found that acetate can increase IFN-β production in alveolar macrophages (AMs), indicating that probiotics can prevent RSV infection in neonatal mice through the microbiota-AM axis. This highlights the potential of probiotics in modulating the microbiota-SCFAs-AM-IFN axis to enhance the body's ability to prevent and fight viral infections. In a recent study (Niu et al., 2023), it was discovered that acetate produced by Bifidobacterium pseudolongum NjM1 can promote the production of IFN-I by macrophages, which plays a protective role against viral infection. Mechanistically, the acetate receptor GPR43 interacts with the nucleotide-binding oligomerization domain-like receptor protein 3 (NLRP3) to promote the aggregation and signal transduction of the mitochondrial antiviral signaling protein (MAVS). The binding of acetate to GPR43 enhances MAVS aggregation, and downstream activation of TANK binding kinase 1 (TBK1) / interferon regulatory factors (IRF3) leads to the production of IFN-I. Furthermore, acetate can reduce the severity of RSV infection and the viral load by regulating the expression of retinoic acid-inducible gene-I (RIG-I). These findings suggest that acetate produced by certain probiotics can modulate the immune response to enhance the body's ability to prevent and fight viral infections.

#### 4.2.2. Effects of interferon on respiratory viral infections and asthma

Sumino et al. (2012) has demonstrated that reduced antiviral interferon response at birth among infants at high risk of asthma and allergic diseases is significantly correlated with increased acute respiratory infections in the following year. Furthermore, research by Rich et al. (2020) indicated that impaired production of type I or type III IFN might be linked to infection-induced asthma exacerbations. Bergauer et al. (2017) confirmed previous reports of impaired interferon response in stable asthma and found that IFN secretion was overactive during asthma exacerbations associated with rhinovirus infection in children. The authors of the paper suggested that although asthmatic children may have impaired immune conditions under normal circumstances, their ability to upregulate IFNs during the acute phase is preserved. Gonzales-van Horn and Farrar (2015) demonstrated that primary human bronchial epithelial cells from asthmatic patients produced lower levels of IFN-λ in response to experimental RV challenge compared to healthy controls. This impaired interferon secretion was associated with an increased incidence of virus-induced asthma exacerbations. Therefore, it is crucial to conduct further studies to determine whether the impaired interferon secretion observed during virus-induced acute asthma attacks is a general phenomenon or if it is limited only to steady-state conditions.

IFN-α has been shown to reduce the number of infiltrating eosinophils in the airway tissues of asthmatic patients in a dose-dependent manner (Kikkawa et al., 2012). Similarly, type III IFN was found to have a comparable effect. When the level of type III IFN was higher, it was associated with a lower number of eosinophils in sputum, lower levels of IL-8 in BALF, and reduced viral load in patients with respiratory infections (Contoli et al., 2006). Furthermore, there is evidence that type I and type III IFNs can inhibit GATA3 expression, restrict the development of Th2 cells and reduce the secretion of type 2 cytokines (Krammer et al., 2021). The presence and magnitude of type 2 immune responses, characterized by eosinophilia and elevated levels of proinflammatory cytokines such as IL-4, IL-5 and IL-13, are believed to be key mechanisms in the development of asthma. On the other hand, type IFN-1 has been shown to negatively regulate the development and activation of human and mouse Th17 cells.

Additionally, interferon indirectly impacts the immune response through the IFN-λ-TSLP axis. Thymic stromal lymphopoietin (TSLP) is an adaptive immunomodulator produced by epithelial cells, which can be induced by IFN-III in mice infected with live attenuated influenza virus. TSLP is an important alarm protein for allergic reactions associated with Th2 inflammation. TSLP has been shown to act as a mediator of eosinophilia in viral infections *in vitro* (Lee et al., 2012). However, its role in eosinophilia *in vivo* has not been well-documented and requires further investigation through additional studies.

Studies have demonstrated that the IFN-γ response is associated with total BALF infiltration and that decreasing IFN-γ production can alleviate airway inflammation and AHR (Zang et al., 2011; Kumar et al., 2012). In addition, IFN-γ can stimulate macrophages to produce IL-27, leading to steroid-resistant airway inflammation and AHR (Kumar et al., 2012). This suggests that targeting the effects of IFN-γ on pulmonary macrophages may be particularly relevant for treating acute exacerbations of steroid-resistant asthma. It is noteworthy that decreased intestinal microbial diversity can lead to increased IFN-γ production, which not only affects the AHR of asthma but also increases the mortality of respiratory viral infections (Grayson et al., 2018).

### 4.3. The impact of vitamin D deficiency, which can be modulated by gut microbes, on respiratory viral infections and asthma

#### 4.3.1. Bidirectional regulation between gut microbiome and vitamin D metabolism

**Figure 4:**
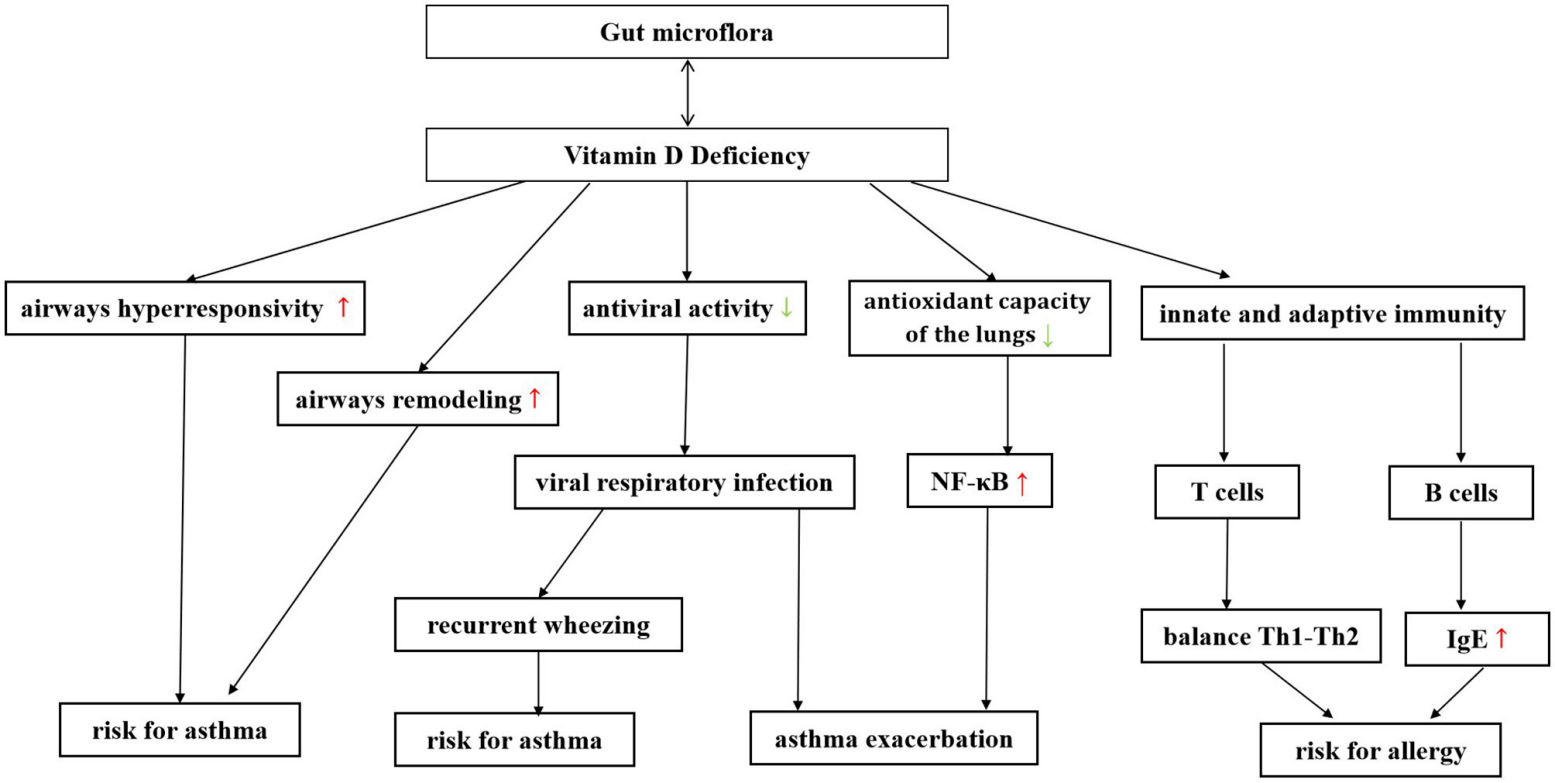
The gut microbiome can be bidirectionally regulated with vitamin D, and supplementation of probiotics can improve vitamin D deficiency in patients with asthma. Vitamin D deficiency has been found to increase the risk of asthma exacerbations through various pathways. A decrease in vitamin D can increase airway hyperresponsiveness and promote airway remodeling. Moreover, a decrease in vitamin D can reduce the body's antiviral ability, making it more susceptible to severe respiratory virus infections, thereby further increasing the risk of asthma and asthma exacerbation. Vitamin D can play a protective role in asthma by reducing oxidative capacity in serum and lung tissue, improving antioxidant capacity, reducing the formation of nitric oxide in serum, and decreasing the expression of nuclear factor kappa B in the lung. Vitamin D deficiency can also induce a shift in the balance between Th1-type and Th2-type cytokines toward Th2 dominance. Vitamin D deficiency can increase the risk of asthma, which is related to its ability to regulate B cell activity and affect IgE production.

Vitamin D (VD) and gut microbiome seem to affect the immune system in respiratory diseases in a variety of similar ways. There may be some interaction and/or synergy between them (Murdaca et al., 2021). Shang and Sun (2017) and Bora et al. (2018) have shown that gut microbiome can alter gut vitamin D metabolism (VDM) and that probiotic supplements can affect circulating VD levels. Meanwhile, people with higher 1,25(OH)2D levels were more likely to have a favorable gut microbiota, especially more butyrate-producing bacteria. There is a bidirectional signal between bacteria and colonic epithelium, and the gut microbiome can also directly affect VDM (Waterhouse et al., 2019). Notably, VD deficiency affects both the occurrence of respiratory infections and the exacerbation of asthma (Confino-Cohen et al., 2014; Ganmaa et al., 2022). Figure 4 summarizes the effect of vitamin deficiency on the increased risk and exacerbation of asthma. This suggests that gut microbiota may play a more complex role in infection and asthma exacerbation by altering VD levels.

#### 4.3.2. Vitamin D can reduce the risk of asthma occurrence and exacerbation by anti-viral infection

Epidemiological studies have demonstrated a significant link between vitamin D deficiency and viral respiratory infections. A cohort study involving children from various countries found that vitamin D supplementation was inversely related to RV infection (Jartti et al., 2021). Furthermore, children with lower respiratory tract infections had notably lower average vitamin D levels than the controls. The incidence and severity of lower respiratory tract infections were also correlated with vitamin D levels (Şişmanlar et al., 2016). Anitua et al. (2022) found that vitamin D supplementation had a minor but positive effect in lowering the risk of one or more acute respiratory syndromes when compared to placebos.

Toll-like receptor 3 (TLR3) is a viral double-stranded RNA recognition receptor that, in viral infections, can exacerbate diseases by inducing tissue damage and facilitating viral dissemination (Wang et al., 2020). Excessive TLR3-mediated inflammation may play a key role in promoting asthma exacerbation and fibrosis, with bronchial smooth muscle cells (BSMCs) being one of the major contributors to asthma airway remodeling (Yang et al., 2017). Plesa et al. (2021) indicate that vitamin D3 can attenuate TLR3 agonist-induced inflammation and fibrotic responses in BSMCs, exerting a protective effect in the development of worsened conditions associated with viral infections. These results support vitamin D3 supplementation in virus-infected asthma patients. This study also found that BSMCs from asthmatic patients were more sensitive to TLR3 agonist (polyI:C) stimulation than those from control subjects. It may indicate a greater propensity for severe reactions in virus-infected asthma patients. In primary bronchial epithelial cells (PBECs) treated with VD, a reduced ability of RV replication and infectivity was observed, suggesting an antiviral effect of VD (Telcian et al., 2017). The effect of VD on respiratory viral infection may be influenced by various factors (such as the frequency, dosage, and duration of vitamin D supplementation; Jartti et al., 2021). Based on the evidence presented above, it can be inferred that vitamin D levels may play a potential role in the association between viral infections and asthma.

#### 4.3.3. Other effects of vitamin D deficiency on asthma exacerbations

VD deficiency is strongly associated with the exacerbation of asthma in both children and adults, as it can influence both innate and adaptive immune responses (Confino-Cohen et al., 2014; Ozkars et al., 2019). VD deficiency may promote the development of AHR and airflow limitation in asthma patients, leading to worsened symptoms (Alyasin et al., 2011; Ozkars et al., 2019). In mouse models, VD deficiency is associated with increased AHR, elevated pro-inflammatory cytokines (IL-10 and TGF-β) in BALF, and reduced Treg cells (Agrawal et al., 2013). Supplementation with vitamin D can reduce inflammation and AHR induced by allergens in sensitized mice (Agrawal et al., 2013; Fischer et al., 2016). *In vitro*, Camargo (2018) have shown that VD deficiency can shift the balance between Th1 and Th2 cytokines toward a Th2-dominant response. de Groot et al. (2015) have also demonstrated that VD supplementation can reduce eosinophilic airway inflammation in non-allergic asthma patients, possibly by restoring the production of IL-10. It is worth noting that there is a U-shaped relationship between serum VD levels and asthma, where both VD deficiency and high VD levels increase the risk of asthma, which may be related to VD's regulation of B cell activity and IgE production (Douros et al., 2017).

In asthma, there is an imbalance between the lung's protective antioxidant system and the generation of oxidative species, leading to an increase in oxidative products and activation of intracellular signaling mechanisms of oxidative/reductive reactions (Chamitava et al., 2020). One such mechanism involves the activation of the nuclear factor kappa B (NF-κB) pathway, which further increases the production of inflammatory molecules (Kirkham and Rahman, 2006). Studies in mice have shown that vitamin D can protect against asthma by reducing oxidative stress in the serum and lung tissue. It also can increase antioxidant capacity, decreasing the formation of nitric oxide in the serum, and reducing the expression of NF-κB p65 in the lungs (Adam-Bonci et al., 2021).

It has been reported (Telcian et al., 2017) that respiratory virus infections can lead to down-regulation of VDR mRNA expression in bronchial epithelial cells. Considering the role of VD deficiency in exacerbating asthma, the decrease in VD receptor expression induced by viral infections may result in a reduction of the protective effect of VD on asthma.

## 5. Conclusion

The regulation of host immunity is highly dependent on gut microbiome. A plethora of data supports the notion that gut microbial dysbiosis may contribute to the development of respiratory infections and asthma. This is particularly relevant in the case of children, where microbial changes in early life can have long-lasting effects on immune system maturation and overall health, ultimately increasing the risk of asthma onset and exacerbation. Respiratory viral infections have been identified as a risk factor for asthma occurrence and exacerbation, and the complex underlying mechanisms warrant further investigation. We postulate that dysbiosis of the gut microbiota may serve as a bridging factor in the development of asthma triggered by respiratory infections. Restoring the homeostasis of the gut microbiome could potentially become a viable strategy for preventing childhood asthma.

## Author contributions

Collection and analysis were performed by YYa, HZ, and SS. The first draft of the manuscript was written by XZ and MH. The conception of the study was put forward by ZX and YYo firstly. All authors contributed to the study's conception and design, commented on previous versions of the manuscript, read, and approved the final manuscript.
